# Supplementary material for: Enzymatic Specificity of Conserved Rho GTPase Deamidases Promotes Invasion of Vibrio parahaemolyticus at the Expense of Infection
Source: mBio. 2022 Jul 7;13(4):e01629-22. doi: 10.1128/mbio.01629-22 (PMC9426531; doi:10.1128/mbio.01629-22)
Supplement: TABLE S1 [file mbio.01629-22-s0004.docx]

**
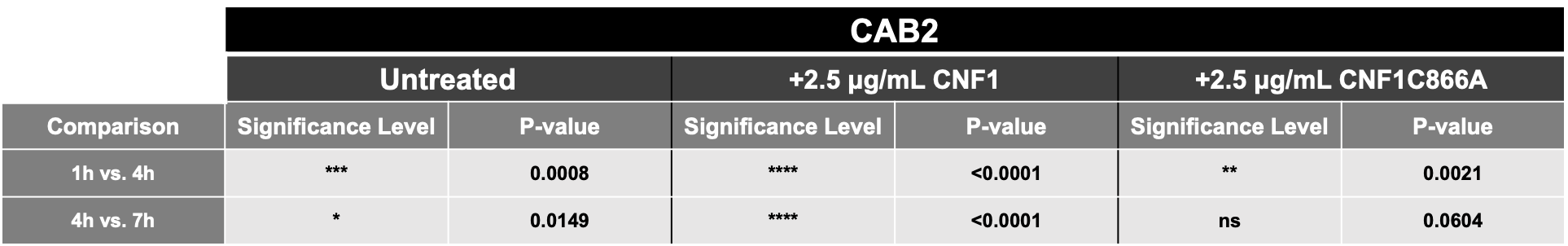
**

**
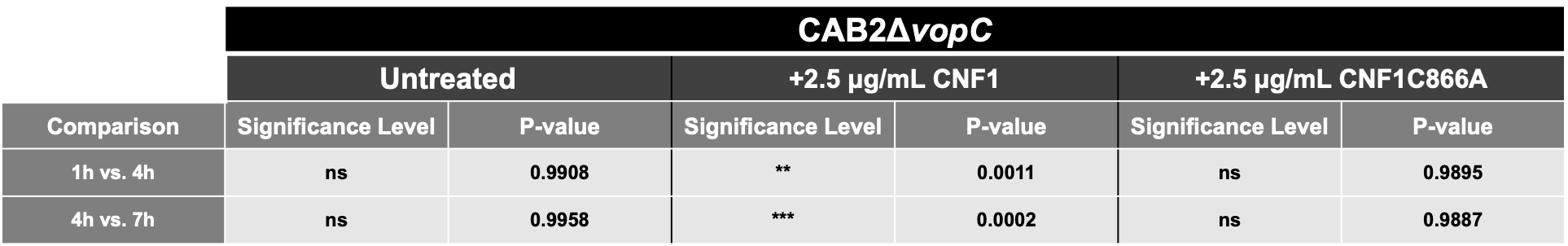
**

**
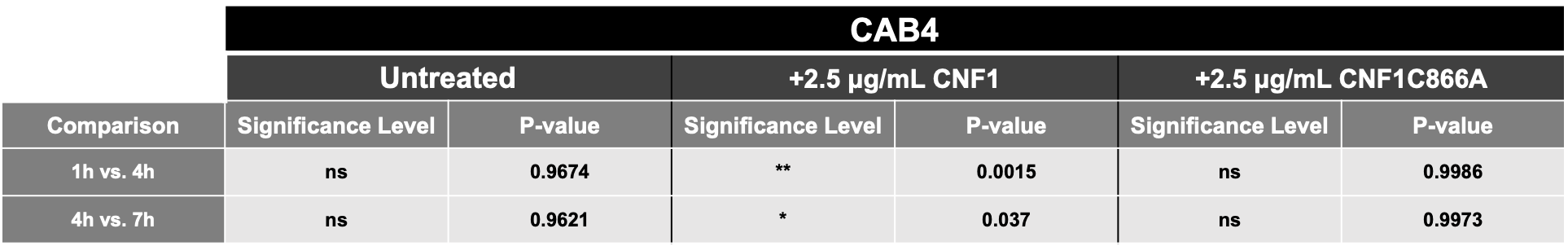
**

**Table S1** Expanded list of gentamicin protection assay statistical comparisons from Figure 3. Briefly**,** gentamicin protection assay comparing intracellular CAB2, CAB2Δ*vopC*, or CAB4 at 1, 4, and 7 hour post-gentamicin application, which proceeded infection and mock, 2.5μg/mL CNF1, or 2.5μg/mL CNF1 C866A application by 2 hours. All infections were conducted at an MOI = 10 Statistical significance measured using a two-way ANOVA with multiple comparisons test (ns, not significant; *, P < 0.05; **, P < 0.005; ***, P < 0.0005; ****, P < 0.00005)
